# Predictors of Percutaneous Catheter Drainage (PCD) after Abdominal Paracentesis Drainage (APD) in Patients with Moderately Severe or Severe Acute Pancreatitis along with Fluid Collections

**DOI:** 10.1371/journal.pone.0115348

**Published:** 2015-02-06

**Authors:** Wei-hui Liu, Tao Wang, Hong-tao Yan, Tao Chen, Chuan Xu, Ping Ye, Ning Zhang, Zheng-cai Liu, Li-jun Tang

**Affiliations:** 1 General Surgery Center, Chengdu Military General Hospital, Chengdu, Sichuan Province, 610083, China; 2 General Surgery Center, Xijing Hospital, Xi’an, Shanxi Province, 710032, China; Oregon State University, UNITED STATES

## Abstract

**Aims:**

Although we previously demonstrated abdominal paracentesis drainage (APD) preceding percutaneous catheter drainage (PCD) as the central step for treating patients with moderately severe (MSAP) or severe acute pancreatitis (SAP), the predictors leading to PCD after APD have not been studied.

**Methods:**

Consecutive patients with MSAP or SAP were recruited between June 2011 and June 2013. As a step-up approach, all patients initially received medical management, later underwent ultrasound-guided APD before PCD, if necessary, followed by endoscopic necrosectomy through the path formed by PCD. APD primarily targeted fluid in the abdominal or pelvic cavities, whereas PCD aimed at (peri)pancreatic fluid.

**Results:**

Of the 92 enrolled patients, 40 were managed with APD alone and 52 received PCD after APD (14 required necrosectomy after initial PCD). The overall mortality was 6.5%. Univariate analysis showed that among the 20 selected parameters, 13 factors significantly affected PCD intervention after APD. Multivariate analysis revealed that infected (peri)pancreatic collections (P = -0.001), maximum extent of necrosis of more than 30% of the pancreas (P = -0.024), size of the largest necrotic peri(pancreatic) collection (P = -0.007), and reduction of (peri)pancreatic fluid collections by <50% after APD (P = -0.008) were all independent predictors of PCD.

**Conclusions:**

Infected (peri)pancreatic collections, a largest necrotic peri(pancreatic) collection of more than 100 ml, and reduction of (peri)pancreatic fluid collections by <50% after APD could effectively predict the need for PCD in the early course of the disease.

## Introduction

Acute pancreatitis (AP) is among the most variable of all known diseases and is one of the first benign disorders to lead to hospital admission [1]. Severe acute pancreatitis (SAP) accounts for 20% of all AP cases, and these severe cases require triage to either aggressive early treatment, transfer to intensive care, or referral to tertiary specialist centres [2, 3]. Morbidity and mortality remain high in SAP. It is widely accepted that operative procedures should be delayed as long as possible to decrease morbidity and mortality. Moreover, minimally invasive retroperitoneal necrosectomy (MINE) and endoscopic transgastric necrosectomy (ETN) are gradually being adopted to replace open necrosectomy (ONE) [4]. In 2010, the New England Journal of Medicine reported that to avoid early intervention (even minimally invasive intervention), a step-up approach is a good option for allowing resuscitation, stabilisation, and demarcation [5].

In recent years, the step-up approach, consisting of percutaneous catheter drainage (PCD) followed, if necessary, by (minimally invasive) necrosectomy, has become an effective option for the treatment of SAP [6]. Additionally, it has been deemed as the standard strategy for the treatment of infected necrotising pancreatitis (INP) [7]. A promising alternative to the step-up approach that is gaining worldwide popularity is endoscopic transluminal drainage (ETD) followed, if necessary, by ETN [8]. The release of the revised Atlanta classification of AP enabled standardised reporting of research and permitted timely updating of the step-up approach [9]. Because infection tends to increase with liquefaction of necrosis, it is sensible to drain fluid collections around necrotic lesions [9]. As a result, a promising minimally invasive alternative is the drainage centred step-up approach, which consists of conservative therapy and drainages (including abdominal paracentesis drainage (APD) and PCD) if necessary followed by endoscopic necrosectomy through the path formed by PCD. This type of staged multidisciplinary step-up approach is an especially optimal treatment for AP with fluid collections [10] because the secondary infection of (peri)pancreatic fluid collections (PPFC) remains the leading cause of mortality in patients with AP [10, 11].

Acute fluid collections and pseudocyst formation are the most frequent complications of AP [12]. The incidence of fluid collections occurs in 30–50% of patients within 48 h of onset of the disease [13]. The majority of these collections are located in the lesser sac and the anterior pararenal space [14, 15]. However, they may track down into the abdominal cavity, pelvis or mediastinum. The current treatments for fluid collections are diverse and depend on accurate interpretations of radiologic tests. Management options include conservative treatment, PCD, open and laparoscopic surgery, and endoscopic drainage [16]. The choice of treatment relies on the correct diagnosis of the type of fluid collection [17]. Owing to recent developments in imaging modalities and various medical and interventional treatments, the incidence, clinical course, and therapeutic outcomes of fluid collections after AP are expected to change. With the exception of application to PPFC, we recently demonstrated the safety and efficacy of sonographically guided APD in removing abdominal or pelvic fluid collections, which, in turn, greatly benefits AP patients [18]. Especially, as a bridge step for further PCD, the application of APD is advocated owing to its less invasive and effective character if performed early enough.

It is well known that a considerable number of AP patients can be treated with PCD without the need for surgical necrosectomy [19]. Additionally, PCD has been shown to be effective for the treatment of factors that lead to surgery after PCD [20]. However, there are currently no reliable criteria to predict which patients may benefit from PCD [20]. In a multivariate logistic regression analysis of clinical and endosonographic parameters, liquid content was shown to be an independent predictor for PCD intervention [21]. A high liquid content in pancreatic necrosis resulted in a 64% accuracy for the prediction of endpoint risk (compared to 2% for solid necrosis). Pancreatic necrotic cavities with high liquid content are associated with a high risk of complications [21]. Nevertheless, the exact predictors for PCD intervention, especially following APD, are still far from clear. In the present study, we reported the factors that indicate the necessity of PCD after APD.

## Methods

### 1 Study objective

The primary aim of this study was to investigate the indicative factors for upgrading to PCD from APD in patients with moderately severe acute pancreatitis (MSAP) or SAP with fluid collections. In this retrospective study, all patients with AP presenting to the PLA Center of General Surgery (Post Graduate Institute of Medical Education Research, China) from June 2011 to June 2013 were included. The diagnosis of MSAP and SAP was based on clinical findings, biochemical parameters, and the computerised tomography severity index score (CTSI) as per the Atlanta Classification [9].

**1.1 Ethics**. All experiments were performed in accordance with clinical study protocols and approved by the Research Care and Ethics Committee at the Chengdu Military General Hospital. The protocol number was SCCT2011–012. The written informed consent was given by participants (or next of kin/caregiver in the case of children) for their clinical records to be used in this retrospective study. If consent was not obtained all the patient records/information was anonymized and de-identified prior to analysis.

**1.2 Inclusion Criteria**. The inclusion criteria were AP with one or more of the following [9]: (1) fluid collections within two weeks of disease onset; (2) single- or multi-organ failure; (3) CTSI > = 7 (initial CT performed within 7 days after the onset of disease.); and (4) acute physiology and chronic health evaluation (APACHE) II score > = 8.

**1.3 Exclusion Criteria**. Cases with the following were excluded: (1) patients without APD interventions; (2) patients who underwent necrosectomy directly after APD without PCD as a bridge therapy; (3) previous percutaneous drainage or surgical necrosectomy during the episode of pancreatitis; (4) previous exploratory laparotomy for acute abdomen and intraoperative diagnosis of AP.

### 2 Indications for interventions

**2.1 Indication for APD**. APD management was introduced to AP patients with a sufficient volume of abdominal or pelvic fluid collections (more than 50 ml). In addition, there should be a feasible pathway for APD under imaging examination.

**2.2 Indications for PCD**. According to others’ criteria [7, 8, 20, 22], most of PCD was performed in patients according to the consensus of AP treating board in our institution. In general, PCD was performed in patients who did not improve with previous APD management in the form of: persistent fever, persistently raised leukocyte count/increasing trend of leukocyte count, worsening or new-onset organ failure, or presence of gas in the pancreatic bed.

**2.3 Indications for necrosectomy**. Indications for necrosectomy included patients who did not improve with previous management, as evidenced by persistent/worsening sepsis after previous management, ongoing or tendentious leukocytosis, persistent/worsening or new-onset organ failure, underway sepsis, inadequate drainage of collections and necrosis, failure to thrive, and presence of ongoing necrosis with bowel complications (e.g., necrosis, uncontrolled fistula, obstruction).

### 3 Management protocols of the novel step-up approach

**Fig 1:**
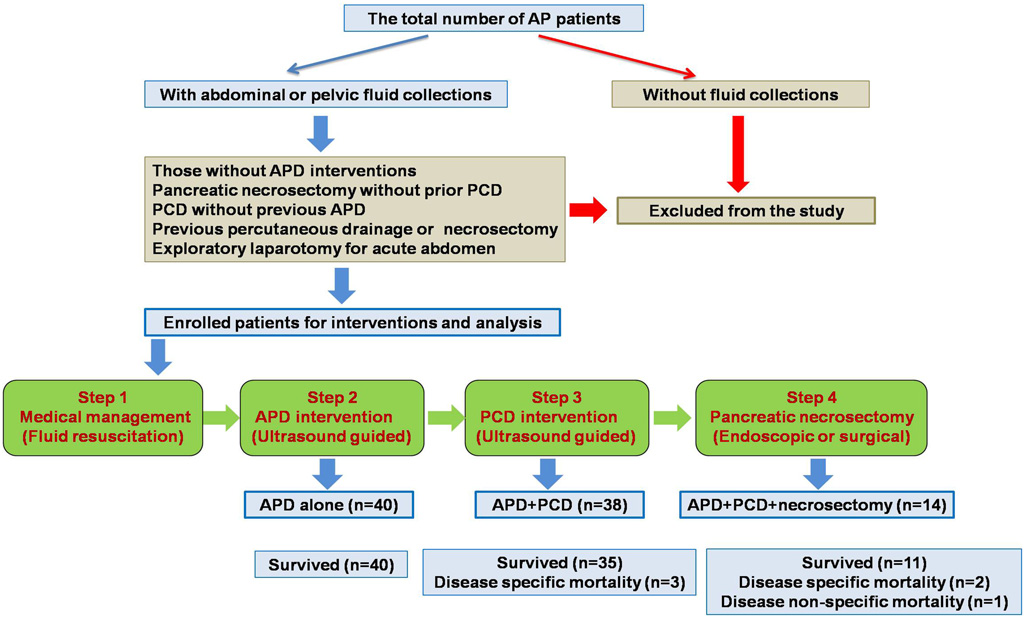
The flow chart of research work. This flow chart generally describes the process of enrolling qualified patients, and introduces the protocol of step-up strategy in managing AP patients.

**Fig 2:**
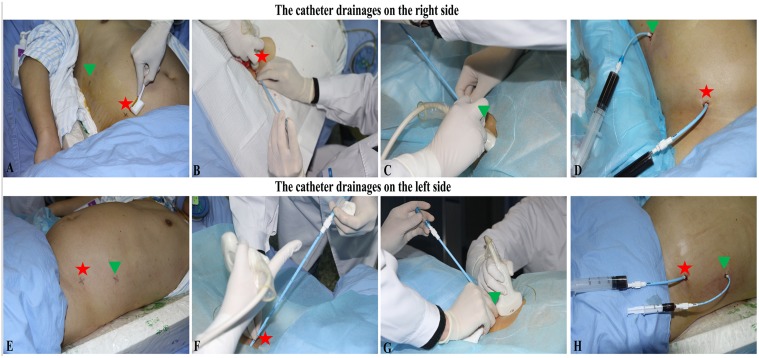
The guidance of catheters placement under APD and PCD. A patient with SAP simultaneously accepted both APD and PCD. Two APDs were as follows: 2 catheters respectively in RPCS or LPCS (for egress of fluid), displayed by red pentagon stars. Meanwhile 2 PCDs was in the (peri)pancreatic region, separately located in RPRR and LPRR, as presented by green triangle pieces. (A) Set the paracentesis points for APD and PCD on the right side; (B) Operate right APD into RPCS; (C) Perform right PCD into RPRR; (D) Confirm fluent drainage through right APD and PCD catheters. (E) Set the paracentesis points for APD and PCD on the left side; (F) Carry out left APD into LPCS; (G) Perform left PCD into LPRR; (H) Make sure fluent drainage under left APD and PCD catheters. RPCS = right paracolic sulci; LPCS = left paracolic sulci; RPRR = right pararenal region; LPRR = left pararenal region.

**3.1 General protocols (Fig. 1)**. All patients initially received conservative treatment including fluid resuscitation, nutritional support, and prophylactic antibiotics by oral ingestion or through a naso-jejunal tube according to need (first step). Parenteral nutrition (PN) was applied if the enteral route was not available or if the daily requirement of the patient was not being met by enteral feeding alone [7, 8, 20, 22]. After conservative therapy, the APD intervention was applied according to indication. APD can be described simply as ultrasound-guided percutaneous puncture and catheterisation as described in our previous study [18]. The third step, referred to ultrasound-guided PCD, was applied to eliminate the liquefied debris and collections in the (peri)pancreas, similar to other reports [19, 23, 24]. For both APD and PCD, if the catheter drainage was not sufficient, placement of additional catheters or repositioning, replacing, or upsizing of catheters was conducted by a professional intervention radiologist. Additionally, the size, number and location of the initial catheters were determined based on the viscosity, quantity and site of collections/necrosis. The route of drainage was planned by means of trans-abdominal ultrasonography, with free-hand technique used for the placement of the catheters into the liquid area in most cases (Fig. 2). Consistent with many other reports [3, 4, 8, 25], in the case of no clinical improvement or the presence of ongoing necrosis with bowel complications, endoscopic or open pancreatic necrosectomy was applied as the fourth step. In our division, minimally invasive retroperitoneal necrosectomy was performed under choledochoscope in patients with drainage that was previously established through the left lumbar access.

Apart from treatment protocols, the following examinations were conducted within 48 hours of admission: hemogram, coagulogram, serum calcium levels, liver and renal function tests, and blood gas analysis. Blood cultures were generally obtained every two weeks after the onset of disease. In addition, once a fever was suspected, an extra blood culture was conducted. Initial contrast-enhanced computed tomography (CECT) was performed within the first week of the onset of disease and repeated every month. A trans-abdominal ultrasound was conducted if necessary. The APACHE II score and CTSI were calculated at the time of admission (averagely within 7 days after the onset of disease).

**Fig 3:**
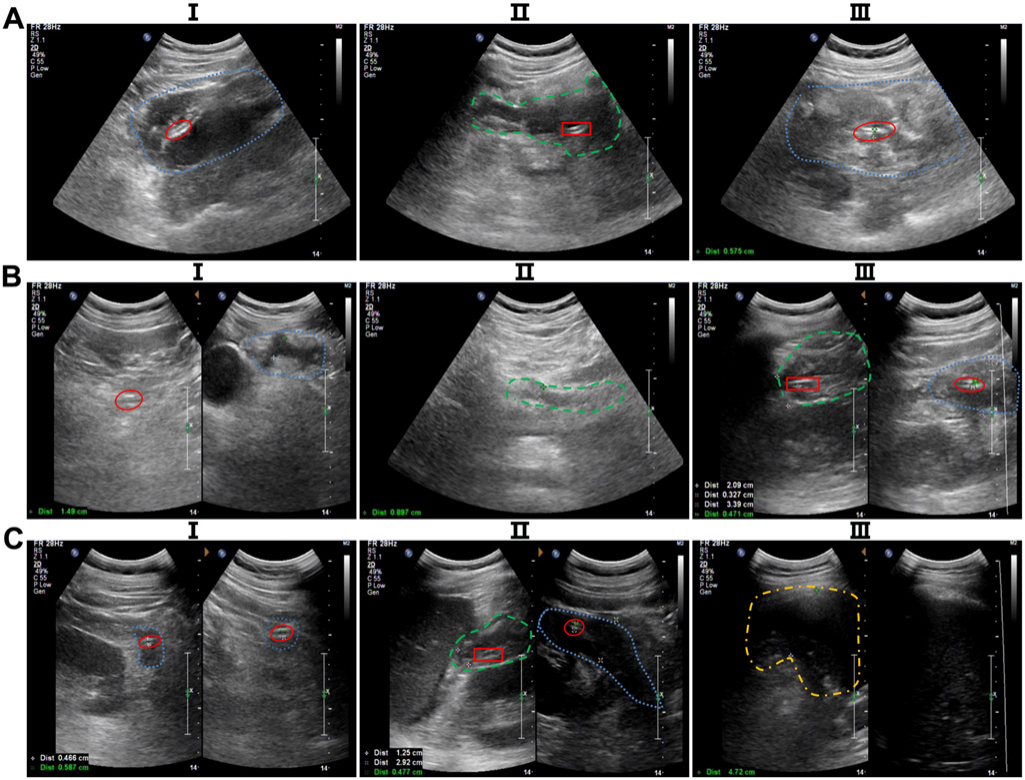
Ultrasonic images of fluid collections and drainage catheters at different time intervals after the interventions. (A) One day after drainage interventions: I, the amount of fluid collection of RPCS was large (blue dashed line) and was extracted by right APD intervention (red ellipse); II, the fluid collection of RPRR was widely distributed (green short line) and was drained by right PCD intervention (red rectangle); III, the amount of fluid collection of LPCS was also large (blue dashed line) and was drained by left APD intervention (red ellipse). (B) Ten days after drainage interventions, I, the amount of fluid collection of RPCS was greatly reduced (blue dashed line), and the fluid was egressed by right APD intervention (red ellipse); II, the fluid collection of the lesser omental bursa is shown by a short green line; III, the fluid collection of LPCS was also lessened in amount (blue dashed line) and was diminished by left APD intervention (red ellipse), and the fluid collection of LPRR decreased substantially (green short line) and was drained by left PCD intervention (red rectangle). (C) At 20 days after drainage interventions, I, the fluid collection of RPCS was almost gone (blue dashed line) and the right APD intervention (red ellipse) remained in place; II, the fluid collection of LPCS was slightly decreased (blue dashed line) and extracted by left APD intervention (red ellipse); meanwhile, the fluid collection of LPRR was greatly reduced (green short line), and the fluid was extracted by left PCD intervention (red rectangle); III, the amount of fluid collection in the left pectoral cavity was large (yellow short and dashed line), and the fluid was drained by extra-catheter intervention; in contrast, there was minimal fluid in the right pectoral cavity. RPCS = right paracolic sulci; LPCS = left paracolic sulci; RPRR = right pararenal region; LPRR = left pararenal region.

**3.2 Indications of catheter extraction**. Patients were instructed about catheter care and irrigation before they were discharged from our institution with catheters in situ. In the following situations, catheters could be extracted: (1) catheter output of less than 10 ml per day of nonpurulent fluid for 2 consecutive days (after adequate flushing and ensuring the patency) with normal lipase levels; (2) no residual collection on a serial CT scan/ultrasonography; or (3) clinical recovery, i.e., no fever, accepting a normal diet, gaining weight, able to carry out routine activities. Notably, the change in the size of the collection was estimated only in patients who had a follow-up CT scan/ultrasonography within 2 weeks after the initial APD or PCD (Fig. 3). The follow-up CT scan/ultrasonography were conducted in the following situations: (1) four days before and one week after each type of intervention; (2) within one month from the last examination; (3) once infection was suspected.

**3.3 Criteria for clinical improvement**. According to the PANTER trial [3], similar criteria were used to define clinical improvement, failure, and procession to the next step. Each step was evaluated 72 hours after intervention and considered successful in cases of clinical improvement. Clinical improvement is defined as improved function of at least two organ systems (i.e., circulatory, pulmonary, or renal) or improvement of 2 out of 3 parameters of infection (i.e., C-reactive protein, leucocytes, or temperature). Clinical failure was defined as the absence of clinical improvement or cases of clinical deterioration. If there was clinical failure at evaluation or at any moment thereafter, the next step or next necrosectomy was performed. Deterioration by infectious causes other than infected necrosis (e.g., a urinary tract infection) was excluded.

### 4 Clinical parameters

**4.1 Primary Endpoints**. Identification of factors that predicted the need for PCD in patients initially treated with APD.

4.2 Secondary Endpoints.

1. Mortality.
2. Proportion of patients requiring PCD after initial APD.
3. Length of intensive care unit (ICU) and hospital stay.
4. Sepsis reversal with APD.
5. Number of interventions required.
6. Catheter-related complications.
7. Morbidity and mortality in patients requiring PCD.
8. Reduction of abdominal or pelvic collections after APD.
9. New occurrence of infection after APD. The diagnosis of infection (infected necrosis) can be suspected by the patient’s clinical course, by the presence of gas within the collection observed on CECT, or by fine needle aspiration (FNA) for culture; however, if PCD is part of the management algorithm, FNA can be replaced by PCD.
10. Size of the largest necrotic peri(pancreatic) collection before PCD.
11. Serum lipase of ≥1000 U/L after APD.

### 5 Statistical analysis

Statistical analyses were performed using the Statistical Product and Service Solutions (SPSS Inc., Chicago, IL, version 17.0 for Windows). Kolmogorov-Smirnov tests of normality were applied to check the normality of data. If the data were normally distributed, the means were compared using Student’s t test between two groups. For comparative analysis of more than two groups, analysis of variance (ANOVA) was used. For skewed data, the Mann-Whitney test was applied. Frequencies or proportions were introduced to describe qualitative and categorical variables. Proportions were compared using the Chi-squared test or Fisher exact test, whichever was applicable. A univariate analysis for predictors was conducted using logistic regression. A multivariate analysis for independent predictors was also performed using logistic regression. All statistical tests were 2-sided and performed at a significance level of *P* < 0.05. The association of potential predictors was determined by simple linear regression. Multiple linear regression was used for adjustment.

## Results

Of the 120 patients with MSAP or SAP admitted to our division between June 2011 and June 2013, 22 were managed with medical therapy only (7 of which lacked enough fluid collections in the abdominal or pelvic cavities for APD interventions) and were excluded from the study. Four patients were excluded from the study for the following reasons: 2 had pancreatic necrosectomy at an index hospital and were referred to us for further management, 1 underwent endoscopic retrograde cholangiopancreatography before admission, and 1 received a laparotomy for acute abdomen and was intraoperatively determined to have SAP. In addition, 2 patients were removed because they received necrosectomy without a previous bridge therapy of PCD. Of the remaining 92 patients recruited in the study, 40 patients were treated with APD alone and 52 received sequential APD and PCD intervention. A total of 14 patients who received sequential APD and PCD interventions required necrosectomy after initial PCD (Fig. 1).

### 1 Demographic Data

The demographic data (age, sex, aetiology) between the groups managed with APD alone and those requiring further PCD were comparable (Table 1). The main aetiology of AP was bile duct problems (48.1% in the APD+PCD group and 50.0% in the APD-alone group), followed by hyperlipidemia (26.9% in the APD+PCD group and 25.0% in the APD-alone group). Compared with the APD-alone group, the APD+PCD group had a slightly lower number of patients with alcohol abuse (20.0% *vs*. 19.2%, respectively; P>0.05). In addition, the APACHE II scores, Ranson scores, and Marshall scores were significantly higher in the APD+PCD group compared with the APD-alone group (*P*<0.05) (Table 1). The mean number of days from the onset of symptoms to discharge from our hospital was also higher in the APD+PCD group than in the APD-alone group (*P*<0.05) (Table 1). The average total cost during hospitalisation was significantly higher in the APD+PCD group than in the APD-alone group (*P*<0.05).

**Table 1:** The characteristics of 92 AP patients enrolled in this study.

Characteristic	APD+PCD group	APD-alone group	P
Number of patients	52	40	
**Demographic data** (mean ± SD)			
Age	48 ± 12.9	50 ± 13.4	0.072
Referral after AP onset (days)	11 ± 8.7	11 ± 7.8	0.615
Etiology			0.812
Gallstone	25 (48.1%)	20 (50.0%)	
hyperlipemia	14 (26.9%)	10 (25.0%)	
Alcohol abuse	10 (19.2%)	8 (20.0%)	
Other	3 (5.8%)	2 (5.0%)	
**Severity scores** (mean± SD)			
Initial APACHⅡscore	15.3 ± 3.47 (10–62)	10.2 ± 2.81 (7–46)	0.003*
Initial CTSI score	8.4 ± 2.38 (6–12)	5.8 ± 2.27 (6–10)	0.005*
Initial Ranson score	3.4 ± 1.27 (1–8)	2.5 ± 1.01 (1–6)	0.024*
Initial Marshall score	4.6 ± 1.76 (3–6)	3.2 ± 0.85 (1–5)	0.014*
**Indexes of medical economics** (median± interquartile range)			
Days in hospital	58.7 ± 25.63	32.1 ± 22.65	0.007*
Days in intensive care unit (ICU)	7.8 ± 4.15	3.2 ± 1.25	0.024*
Total cost during hospitalization (Dollars)	11214.6 ± 4525.35	6012.7 ± 4126.45	0.003*

Abbreviations: APD = abdominal paracentesis drainage; PCD = percutaneous catheter drainage; AP = acute pancreatitis; APACHE II = acute physiology and chronic health evaluation II; CTSI = computerized tomography severity index; APD+PCD group = patients in this group treated with APD ahead of PCD; APD-alone group = patients in this group treated only with APD.* Significant difference.

### 2 Parameters between the APD-alone group and the APD+PCD group

**2.1 Clinical and laboratory parameters**. The clinical and laboratory parameters were collected seven days after initial APD application (approximately 20 days from the onset of AP) in both groups. The APACHE II scores, Ranson scores, and Marshall scores were higher in the APD+PCD group compared with the APD-alone group (*P*<0.05) (Table 2). As to the maximum extent of pancreatic necrosis during the hospital stay, we found that the APD+PCD group had a higher number of patients with more than 30% necrosis compared with the APD-alone group (Table 2). The most common site of extrapancreatic necrosis was the left anterior pararenal region. We observed that the APD+PCD group had a significantly higher amount of fluid collections compared with the APD-alone group (Table 2).

**Table 2:** The laboratory and clinical parameters between two groups.

	APD+PCD group	APD-alone group	P
Number of patients	52	40	
Maximum extent of necrosis during the stay			0.015*
Less than 30%	7 (13.5%)	10 (25.0%)	
30%–50%	24 (46.2%)	25 (62.5%)	
More than 50%	21 (40.4%)	5 (12.5%)	
Maximum extent of fluid collections			0.002*
40–100 ml	7 (13.5%)	6 (15.0%)	
100–200 ml	25 (57.7%)	30 (75.0%)	
More than 200ml	20 (38.5%)	4 (10.0%)	
Laboratory parameters (onset of AP)			
C-reaction protein (CRP) (mg/L)	152.5 ± 23.45	125.5 ± 21.76	0.042*
IL-6 (pg/L)	385.5 ± 72.16	215.5 ± 42.25	0.015*
IL-10 (pg/L)	150.7 ± 45.62	103.4 ± 24.32	0.021*
TNF-α (pg/L)	20.4 ± 6.18	19.4 ± 4.36	0.375
Laboratory parameters (after APD)			
C-reaction protein (CRP) (mg/L)	72.6 ± 18.45	60.7 ± 17.35	0.037*
IL-6 (pg/L)	90.4 ± 21.26	87.6 ± 20.52	0.078
IL-10 (pg/L)	40.3 ± 10.17	38.6 ± 10.03	0.120
TNF-α (pg/L)	13.1 ± 4.57	12.7 ± 4.26	0.306
Severity scores (after APD)			
APACHⅡscore (mean± SD)	10.8 ± 2.37 (3–36)	6.8 ± 2.15 (3–22)	0.004*
Ranson score (mean± SD)	2.8 ± 1.06 (1–8)	2.4 ± 0.88 (1–6)	0.025*
Marshall score (mean± SD)	3.3 ± 0.72 (1–8)	2.1 ± 0.58 (1–6)	0.007*

Abbreviations: APD = abdominal paracentesis drainage; PCD = percutaneous catheter drainage; AP = severe acute pancreatitis; APACHE II = acute physiology and chronic health evaluation II; CTSI = computerized tomography severity index; APD+PCD group = patients in this group treated with APD ahead of PCD; APD-alone group = patients in this group treated only with APD.* Significant difference.

Although the levels of initial laboratory parameters (CRP, IL-6, IL-10) were significantly higher in the APD+PCD group than in the APD-alone group (*P*<0.05), APD led to a large decrease in those laboratory parameters in both groups (Table 2). For example, the mean level of serum CRP was reduced by 53% in the APD+PCD group (from 152.5 ± 23.45 mg/L to 72.6 ± 18.45 mg/L) and by 52% in the APD-alone group (from 125.5 ± 21.76 mg/L to 60.7 ± 17.35 mg/L). These results demonstrated that a large number of inflammatory factors were eliminated through drainage of the seroperitoneum by APD in both groups.

**2.2 Infection-related parameters**. More patients in the APD+PCD group (44 of 52) had polymicrobial infections compared with the APD-alone group (30 of 40). *Escherichia coli* (*E*. *coli*) was the most common bacterial isolate in this study, with a significantly higher incidence in the APD+PCD group compared with the APD-alone group (*P*<0.05) (Table 3). At admission, the mean white blood cell (WBC) count in the APD+PCD group (14 ± 2.2 ×10E9/L) was higher than that of the APD-alone group (11 ± 1.6 ×10E9/L); meanwhile, WBC recovery took longer in the patients in the APD+PCD group than in the APD-alone group (*P*<0.05). Moreover, the incidence of sepsis was slightly higher in the APD+PCD group (21 of 52, 40%) compared with the APD-alone group (12 of 40, 30%). It took more time for the patients in the APD+PCD group (23.5 ± 2.21 days) to gain sepsis reversal than for the APD-alone group (15.6 ± 1.88 days) (*P*<0.05) (Table 3).

**Table 3:** The infectious parameters between two groups.

	APD+PCD group	APD-alone group	P
Number of patients	52	40	
Incidence of infections			0.068
Polymicrobial infections	44 (84.6%)	30 (75.0%)	
Monomicrobial infections	5 (9.6%)	4 (10.0%)	
No infection	3 (5.8%)	6 (15.0%)	
WBC count at admission (×10E9/L)	14 ± 2.2	11 ± 1.6	0.036*
The recovery of WBC (days)	30.6 ± 12.18	20.2 ± 9.35	0.007*
The incidence of bacteremia	32 (61.5%))	20 (50.0%)	0.006*
The recovery of bacteremia	19.2 ± 6.74	12.2 ± 4.96	0.015*
The incidence of sepsis	21 (40.4%)	12 (30.0%)	0.021*
Time for sepsis reversal (days)	23.5 ± 2.21	15.6 ± 1.88	0.017*

Abbreviations: APD = abdominal paracentesis drainage; PCD = percutaneous catheter drainage; APD+PCD group = patients in this group treated with APD ahead of PCD; APD-alone group = patients in this group treated only with APD; WBC = white blood cell.* Significant difference.

**2.3 Organ failure**. Based on the modified Marshall scoring system, the parameters of organ failure were collected and analysed [26]. The frequency of organ failure was much higher in the APD+PCD group than in the APD-alone group (*P*<0.05) (Table 4). Additionally, there were significant differences in the mean number of failed organs and the multiple organ failure (MOF) rate between the APD+PCD group and the APD-alone group (*P*<0.05). The mean duration of organ failure in the APD+PCD group was also higher than that of the APD-alone group (*P*<0.05) (Table 4).

**Table 4:** The organ failure related parameters between two groups.

	APD+PCD group	APD-alone group	P
Number of patients	52	40	
Number of organs failed per patient			
Mean ± SD	1.5 ± 1.06	0.7 ± 0.68	0.014*
Median (range)	2 (0–5)	1 (0–5)	
Organs failure			0.021*
No organ failure	10 (19.2%)	12 (30.0%)	
Single-organ failure	18 (34.6%)	20 (50.0%)	
Multiple-organ failure	24 (46.2%)	8 (20.0%)	
Duration of organ failure (days)	25.8 ± 5.16	16.8 ± 3.24	< 0.001*
Reversal of organ failure after APD	16/42 (28.6%)	14/28 (50.0%)	< 0.001*

Abbreviations: APD = abdominal paracentesis drainage; PCD = percutaneous catheter drainage; APD+PCD group = patients in this group treated with APD ahead of PCD; APD-alone group = patients in this group treated only with APD.* Significant difference.

### 3 Parameters between the PCD success group and PCD failure group

The PCD success group included patients with clinical improvements after initial PCD, and the PCD failure group referred to those without clinical improvements after initial PCD (e.g., those who received further necrosectomy, had major complications, or died). The APACHE II scores, Ranson scores, and Marshall scores were significantly higher in the PCD failure group in comparison to the PCD success group (*P*<0.05) (Table 5). We found that the PCD failure group had a higher number of patients with more than 30% necrosis compared with the PCD success group (Table 5). Notably, the maximum extent of fluid collections during the patient stay was much larger in the PCD failure group than in the PCD success group (Table 5). The size of the necrotic collection in the PCD success group decreased by a median of 85% (range, 56–90%) within a median of 20 days after PCD. In contrast, the collection size in the PCD failure group was reduced only by a median of 21% (range, 12–39%) after PCD. The duration between the onset of symptoms and the first PCD insertion was significantly shorter in the PCD failure group than in the PCD success group, whereas the median duration of percutaneous drainage in the patients who failed PCD was significantly longer than in patients who were treated successfully with this technique, 51 versus 23 days. The ICU stay during the entire course, and hospital stay were significantly higher in the PCD failure group than in the PCD success group (Table 5).

**Table 5:** The comparison of related parameters between PCD success and failure groups.

	PCD success group	PCD failure group	P
Number of patients	35	17	
**Severity scores** (mean ± SD)			
Initial APACHⅡscore	17.4 ± 4.12 (10–62)	13.5 ± 3.16 (10–50)	0.015*
Initial CTSI score	8.9 ± 2.64 (6–12)	7.2 ± 2.32 (6–10)	0.021*
Initial Ranson score	4.2 ± 1.56 (1–8)	3.0 ± 1.12 (1–6)	0.006*
Initial Marshall score	5.8 ± 1.82 (3–6)	4.0 ± 1.35 (3–5)	0.007*
Maximum extent of necrosis			0.004*
Less than 30%	7 (20.0%)	0 (0.0%)	
30%–50%	17 (48.6%)	7 (41.2%)	
More than 50%	11 (31.4%)	10 (58.8%)	
Extent of fluid collections			<0.001*
40–100 ml	7 (20.0%)	0 (0.0%)	
100–200 ml	22 (62.9%)	3 (17.6%)	
More than 200ml	6 (17.1%)	14 (82.4%)	
Reduction of fluid collections	85.6% (56–90%)	21.8% (12.2–39%)	<0.001*
**Interval from AP onset to initial PCD** (days)	34.6 ± 6.42	22.4 ± 5.16	0.002*
Duration of PCD	23.6 ± 4.31	51.2 ± 7.23	0.015*
Mortality	0 (0.0%)	6 (35.3%)	<0.001*
**Indexes of medical economics** (median± interquartile range)			
Days in hospital	43.4 ± 16.58	71.2 ± 21.58	0.003*
Days in intensive care unit (ICU)	5.8 ± 2.16	12.4 ± 3.76	0.002*
Total cost during hospitalization (Dollars)	8147.5 ± 4525.35	18217.8 ± 6215.68	<0.001*

Abbreviations: APD = abdominal paracentesis drainage; PCD = percutaneous catheter drainage; AP = acute pancreatitis; APACHE II = acute physiology and chronic health evaluation II; CTSI = computerized tomography severity index; PCD success group = patients in this group survived without further interventions after PCD; PCD failure group = patients in this group died or needing further interventions after PCD.* Significant difference.

### 4 Characteristics of APD

The interval between the onset of symptoms and the first APD insertion was similar in the APD-alone group and the APD+PCD group. The total number of catheters used under APD was 87 in 52 patients of the APD+PCD group, and 68 in 40 patients in the APD-alone group. The most frequently used size of catheter in both groups was 12 Fr. The median duration of APD in the APD+PCD group was 21.3 days compared to 15.2 days in the APD-alone group. Overall, the total number of interventional procedures (repositioning, replacements, flushing, upsizing, and additional catheter placement under image guidance) was much more in the APD+PCD group than in the APD-alone group (Table 6).

**Table 6:** The detailed information of APD interventions between groups.

	APD+PCD group	APD-alone group	P
Number of patients	52	40	
Details for APD intervention			
Interval between the onset of symptoms and the first APD insertion (days)	11.4 ± 3.27	10.8 ± 3.15	0.126
Number of APD catheters per patient			
Mean ± SD	1.7 ± 0.52	1.7 ± 0.48	0.527
Median (range)	2 (1–4)	2 (1–4)	
APD catheter duration (days)			< 0.001*
Mean ± SD	21.3 ± 6.74	15.2 ± 5.17	
Median (range)	25 (6–126)	17 (5–48)	
Total APD procedures per patient			
Mean ± SD	4.5 ± 2.31	4.7 ± 2.46	0.146
Median (range)	5 (1–8)	5 (1–8)	

Abbreviations: APD = abdominal paracentesis drainage; PCD = percutaneous catheter drainage; APD+PCD group = patients in this group treated with APD ahead of PCD; APD-alone group = patients in this group treated only with APD.* Significant difference.

### 5 Characteristics of PCD

A total of 52 patients underwent PCD, including 38 in the APD+PCD group and 14 in the necrosectomy group. For these 52 patients, the total number of catheters used was 82. The median duration of PCD was significantly higher in the APD+PCD group compared to the necrosectomy group. In addition, the number of interventional procedures (repositioning, replacements, flushing, upsizing, and additional catheter placement under image guidance) in the 2 groups was comparable (Table 7). The catheter diameter under PCD ranged from 8 to 16 Fr. The most common size and maximum diameter of initial PCD catheter was 14 Fr. Catheters of 16 or 20 Fr were also used according to need (Table 7).

**Table 7:** The detailed information of PCD interventions.

	APD+PCD group	Necrosectomy group	P
Number of patients	38	14	
Details for PCD intervention			
Interval between the onset of symptoms and the first PCD insertion (days)	32.8 ± 5.78	32.1 ± 5.84	0.215
Number of PCD catheters per patient			
Mean ± SD	1.5 ± 0.38	1.7 ± 0.41	0.073
Median (range)	1 (1–4)	1 (1–4)	
Total PCD procedures per patient			
Mean ± SD	6.0 ± 2.81	5.7 ± 2.58	0.247
Median (range)	6 (1–12)	5 (1–12)	
PCD catheter duration (days)			< 0.001*
Mean ± SD	41.3 ± 30.32	24.1 ± 18.74	
Median (range)	40 (6–254)	21 (11–172)	
Site of PCD			0.068
Pancreatic region	28	10	
Left pararenal region (LPRR)	16	7	
Right pararenal region (RPRR)	9	6	
Right subhepatic region	4	2	
Mortality due to PCD or drains	0	0	-
Morbidity due to PCD or drains	9 (23.7%)	6 (42.9%)	0.012*
Disease-specific mortality	3 (7.9%)	3 (21.4%)	0.005*

Abbreviations: APD = abdominal paracentesis drainage; PCD = percutaneous catheter drainage; APD+PCD group = patients in this group treated firstly with APD and following by PCD; necrosectomy group = patients in this group treated sequentially with APD, PCD and necrosectomy. * Significant difference.

### 6 Endpoints

The mortality rate in the APD-alone group was 0 out of 40 patients (0.0%); in contrast, the mortality rate in the APD+PCD group (6 of 52 patients, 11.5%) was significantly higher (*P*<0.05). The causes of death in the patients with disease-specific mortality were mainly from MOF and severe infection. Of the 6 patients who died of disease, 3 developed MOF together with persistent/worsening sepsis, 2 suffered from ongoing sepsis with MOF, and 1 contracted enterocutaneous fistulas with MOF. After APD interventions, there were 36 patients in the APD-alone group who achieved sepsis reversal (90%), while only 16 patients in the APD+PCD group achieved sepsis reversal (31%). The indications for further PCD in these 16 patients were failure to thrive in 6, new onset of infection in 6, and large area of (peri)pancreatic fluid collections in 4. There was no mortality due to APD per se, and all the APD-related complications were managed with simple graded withdrawal of the APDs. In all of the recruited patients, APD also led to organ failure reversal in 16 out of 48 patients with organ failure (33.3%). This led to the avoidance of further PCD in 10 out of 16 patients (62.5%); in contrast, in the case of patients who showed no organ failure reversal (n = 32), we observed that 28 out of 32 underwent PCD (Table 8).

**Table 8:** The main endpoints in this study.

	APD+PCD group	APD-alone group	P
Number of patients	52	40	
Mortality	6 (11.5%)	0 (0.0%)	<0.001*
Disease specific mortality	6 (11.5%)	0 (0.0%)	
Non- Disease specific mortality	0 (0.0%)	0 (0.0%)	
The causes of death			<0.001*
MOF with persistent/worsening sepsis	3/6 (50.0%)	0 (0.0%)	
MOF with ongoing sepsis	2/6 (33.3%)	0 (0.0%)	
MOF with enterocutaneous fistulas	1 (16.7%)	0 (0.0%)	
Sepsis reversal after APD	16 (30.7%)	36 (90.0%)	<0.001*
Organ failure reversal after APD	6/34 (17.6%)	10/14 (71.4%)	<0.001*

Abbreviations: APD = abdominal paracentesis drainage; PCD = percutaneous catheter drainage; APD+PCD group = patients in this group treated with APD ahead of PCD; APD-alone group = patients in this group treated only with APD.* Significant difference.

### 7 Factors Predicting PCD Intervention

Among the 20 factors studied, both in MSAP and SAP patients 13 were predictive of PCD intervention in the univariate analysis (Table 9), as follows: initial APACHE II score, size of the largest necrotic peri(pancreatic) collection, (suspected) infected (peri)pancreatic collections, symptomatic sterile (peri)pancreatic collections, sepsis reversal by APD within a week, number of failed organs, organ failure within a week of the onset of disease, number of bacteria isolated per patient, CTSI score at admission, parenteral nutrition requirement after APD intervention, serum lipase of ≥1000 U/L after APD, maximum extent of necrosis of more than 30% of the pancreas, and reduction of (peri)pancreatic fluid collections by <50% after APD.

**Table 9:** The factors predicting PCD after previous APD intervention.

	PCD intervention	
	Odds ratio (lower-upper)	P
Univariate Analysis in MSAP and SAP patients		
Initial APACHE II score	1.3 (1.0–1.5)	0.007*
Size of the largest necrotic peri(pancreatic) collection	3.5 (1.2–6.7)	0.012*
(Suspected) infected (peri)pancreatic collections	1.6 (1.5–1.8)	<0.001*
Symptomatic sterile (peri)pancreatic colletions	3.4 (1.3–9.1)	0.009*
Sepsis reversal by APD within a week	6.4 (1.4–21.6)	0.017*
Number of organs failed	3.8 (2.5–7.1)	0.021*
Organ failure within a week of the onset of disease	2.1 (0.8–5.7)	0.036*
Number of bacteria isolated per patient	2.7 (1.3–6.2)	0.005*
CTSI score at admission	1.3 (1.1–2.1)	0.034*
Parenteral nutrition requirement after APD intervention	0.9 (0.5–2.3)	0.041*
Serum lipase of ≥1000U/L after APD	0.9 (0.7–1.1)	0.009*
Maximum extent of more than 30% of the pancreatic necrosis	1.7 (1.2–3.1)	0.013*
Reduction of (peri)pancreatic fluid collections by <50% after APD	2.3 (1.8–11.2)	0.032*
Sex	2.1 (0.7–7.1)	0.315
Age	0.93 (0.87–1.0)	0.189
Alcohol abuse	0.87 (0.51–1.4)	0.617
Gall stones	0.67 (0.25–1.6)	0.341
Hyperlipemia	0.92 (0.43–1.7)	0.105
C-reactive protein	0.96 (0.84–1.0)	0.126
IL-10	0.78 (0.47–1.8)	0.417
Multivariate Analysis in SAP patients		
Model Ⅰ		
Size of the largest necrotic peri(pancreatic) collection	3.7 (2.7–5.4)	0.002*
(Suspected) infected (peri)pancreatic collections	1.2 (1.1–1.3)	<0.001*
Symptomatic sterile (peri)pancreatic colletions	3.1 (1.2–5.3)	0.120
Sepsis reversal by APD within a week	2.3 (2.1–4.3)	0.055
Parenteral nutrition requirement after APD intervention	1.9 (1.1–3.1)	0.342
Serum lipase of ≥1000U/L after APD	1.7 (0.9–2.7)	0.217
Maximum extent of necrosis of more than 30% of the pancreas	2.5 (2.1–3.2)	0.016*
Reduction of (peri)pancreatic fluid collections by <50% after APD	4.6 (3.1–8.2)	0.002*
Model Ⅱ		
Size of the largest necrotic peri(pancreatic) collection	3.8 (3.1–5.7)	0.003*
(Suspected) infected (peri)pancreatic collections	1.2 (1.1–1.4)	<0.001*
Symptomatic sterile (peri)pancreatic colletions	2.9 (1.2–6.3)	0.248
Sepsis reversal by APD within a week	2.8 (2.4–6.6)	0.064
Maximum extent of more than 30% of the pancreatic necrosis	2.5 (2.2–3.8)	0.005*
Reduction of (peri)pancreatic fluid collections by <50% after APD	4.8 (3.8–7.6)	0.001*
Multivariate Analysis in MSAP patients		
Model Ⅰ		
Size of the largest necrotic peri(pancreatic) collection	2.3 (1.7–3.4)	0.015*
(Suspected) infected (peri)pancreatic collections	1.0 (0.9–1.2)	0.012*
Symptomatic sterile (peri)pancreatic colletions	6.7 (4.2–14.3)	0.028*
Sepsis reversal by APD within a week	3.7 (3.1–7.2)	0.015*
Parenteral nutrition requirement after APD intervention	1.2 (0.8–2.1)	0.375
Serum lipase of ≥1000U/L after APD	0.9 (0.6–1.7)	0.272
Maximum extent of necrosis of more than 30% of the pancreas	1.7 (1.4–2.2)	0.023*
Reduction of (peri)pancreatic fluid collections by <50% after APD	2.4 (2.1–4.2)	0.018*
Model Ⅱ		
Size of the largest necrotic peri(pancreatic) collection	2.3 (1.8–3.7)	0.018*
(Suspected) infected (peri)pancreatic collections	1.1 (1.0–1.2)	0.014*
Symptomatic sterile (peri)pancreatic colletions	6.7 (4.3–14.3)	0.036*
Sepsis reversal by APD within a week	4.6 (3.4–8.6)	0.017*
Maximum extent of more than 30% of the pancreatic necrosis	1.7 (1.2–2.9)	0.028*
Reduction of (peri)pancreatic fluid collections by <50% after APD	2.5 (1.8–4.6)	0.021*

Abbreviations: APD = abdominal paracentesis drainage; PCD = percutaneous catheter drainage; MSAP = moderately severe acute pancreatitis; SAP = severe acute pancreatitis. * Significant difference.

**Fig 4:**
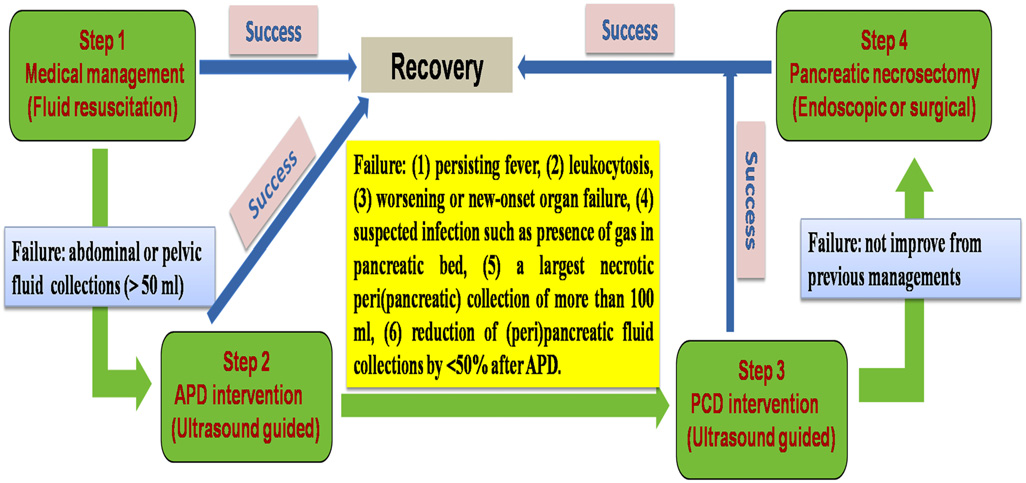
The indication of each intervention in the newly established step-up approach when treating AP patients in this study. Especially, based on our own experience and predicted factors in this study, the indication for escalating from APD to PCD was highlighted.

The number of recruited patients allowed 8 includable parameters in the multivariate analysis. Thus, to avoid redundant parameters in the multivariate analysis, we crossly selected 6 and 8 parameters in two separate models. The multivariate analysis with a Cox proportional hazards model was separately performed in MSAP and SAP patients, using 2 different models (infected (peri)pancreatic collections, size of the largest necrotic peri(pancreatic) collection, reversal of sepsis within a week of APD, reduction of (peri)pancreatic fluid collections by <50% after APD, and maximum extent of necrosis of more than 30% of the pancreas), with the inclusion of the clinicopathological factors that were found to be significant on univariate analysis. Initial APACHE II score, CTSI score at admission, number of failed organs, and organ failure within a week of the onset of disease were not used as dependent variables in the multivariate analysis to avoid confounding, although they were significant factors in the univariate analysis. Infected (peri)pancreatic collections (P = -0.001), maximum extent of necrosis of more than 30% of the pancreas (P = -0.016), size of the largest necrotic peri(pancreatic) collection (P = -0.002), and reduction of (peri)pancreatic fluid collections by <50% after APD (P = -0.001) remained independent predictors of PCD in both MSAP and SAP patients (Table 9). Using a receiver operating characteristic (ROC) curve, we found that infected (peri)pancreatic collections had the best ability to predict PCD with a sensitivity of 95.6% and a specificity of 79.6%, followed by the largest necrotic peri(pancreatic) collection of more than 100 ml with a sensitivity of 88.7% and a specificity of 78.2%. Additionally, reduction of (peri)pancreatic fluid collections by <50% after APD also could predict PCD with a high sensitivity (86.5%) and specificity (76.8%). Based on our own experience and predicted factors in this study, we also depicted a chart (Fig. 4) for escalating the therapy from APD to PCD, which included the following aspects: (1) persisting fever, (2) leukocytosis, (3) worsening or new-onset organ failure, (4) suspected infection such as presence of gas in pancreatic bed, (5) a largest necrotic peri(pancreatic) collection of more than 100 ml, (6) reduction of (peri)pancreatic fluid collections by <50% after APD.

## Discussion

In recent years, the step-up approach has gradually become the standard strategy for treating patients with AP. One criticism of the step-up approach has been that it is applicable for a select group of patients in which necrosis is liquefied and has minimal debris. To extend the application of the step-up approach and to avoid major injury, many researchers are engaged in improving each composite step. Recently, minimally invasive methods of pancreatic necrosectomy have also been developed, including endoscopic [27] and laparoscopic [3, 28] approaches to the necrosis instead of surgical necrosectomy. In addition to image-guided PCD, retroperitoneal laparoscopic drainage is an effective method with minor injury [29]. Combination therapy involving PCD plus endoscopic necrosectomy is associated with good long-term outcomes [22, 30].

Based on the clearance of fluid collections released by the revised Atlanta classification of AP [9], we utilised a novel step-up approach with the addition of APD to better address fluid collections [18]. In our institution, ultrasound-guided APD was used for the early treatment of AP cases with acceptably low morbidity and mortality, which may be the definitive treatment and a bridge management to PCD. In our series, both APD and PCD were performed under ultrasound guidance. Ultrasound-guided drainage is a technique that does not include radiation hazards and has the advantage of real-time imaging. The prerequisite for safe retroperitoneal catheter drainage under sonographic guidance is the extension of the peripancreatic collections at least as far as the lower pole of the kidney. Furthermore, this approach is ideally suited for subsequent minimally invasive pancreatic necrosectomy. However, normal ultrasound-guided drainage is more operator-dependent and provides a worse visualisation of the retroperitoneal space, especially in obese patients. Endoscopic ultrasonography (EUS) is one of the most accurate imaging tools for the pancreas and also provides real-time visualisation of the upper gastrointestinal tract. Meanwhile the close proximity of the endoscopic ultrasound probe to the pancreas results in high spatial resolution that is superior to that of CT and magnetic resonance imaging [31]. Essentially, EUS should be used instead of common ultrasound to guide both APD and PCD to achieve better clinical results.

Since the technique of PCD was first introduced by Freeny *et al*. [32] for the treatment of INP, PCD has been the definitive treatment in approximately one-third of the patients with infected necrosis [3]. By performing drainage of “infected fluid under pressure”, the clinical condition may improve, and the necrotic tissue may be successfully remedied by the patient’s immune system [33]. Except for drainage of infected fluid collections, as (peri)pancreatic fluid is a risk factor for bacterial growth [34], PCD might dramatically reduce fluid collections and prevent partial infection [35].This is evidenced by the fact that patients who did not survive had used PCD for a longer time compared to those who survived. Additionally, although the inclusion of PCD in the step-up approach alters the natural course of AP due to its ability to reverse sepsis and organ failure, PCD used for this indication has often been criticised for its poor ability to remove solid debris. It remains unclear which subset of patients among those managed by the step-up approach would require PCD. Additionally, the timing of PCD remains an issue that requires attention. There are no definitive criteria that allow the prediction of which patients with AP are likely to benefit from PCD. Horvath et al. [36] found that a reduction in collection size of 75% at 10–14 days after PCD predicted the success of PCD with 100% accuracy. In our series, PCD was successful when the collection size decreased by a median of 80%, whereas a negligible reduction in collection size (12–39%) predicted failure of PCD. Specifically, PCD appears to be best suited for patients with liquefied necrosis. That is, this procedure should be delayed as long as possible to allow adequate liquefaction of the necrotic debris [37].

In the present study, we aimed to understand the circumstances under which PCD benefits patients with AP, especially after APD interventions. After the bridging value of inserted APD for later PCD in the novel step-up approach was demonstrated in our previous study [18], the present study was conducted to identify predictive factors of PCD management after APD intervention. In the univariate analysis, the significant factors included initial APACHE II score, size of the largest necrotic peri(pancreatic) collection, (suspected) infected (peri)pancreatic collections, symptomatic sterile (peri)pancreatic collections, sepsis reversal by APD within a week, number of failed organs, organ failure within a week of the onset of disease, number of bacteria isolated per patient, CTSI score at admission, parenteral nutrition requirement after APD intervention, serum lipase of ≥1000 U/L after APD, maximum extent of necrosis of more than 30% of the pancreas, and reduction of (peri)pancreatic fluid collections by <50% after APD. Of these 13 significant factors in the univariate analysis, 4 factors can be used early in the course of disease to predict the outcome of PCD or management with APD alone: infected (peri)pancreatic collections, size of the largest necrotic peri(pancreatic) collection, reduction of (peri)pancreatic fluid collections by <50% after APD and maximum extent of necrosis of more than 30% of the pancreas. Of these 4 significant factors that are predictive of PCD, two parameters—size of the largest necrotic peri(pancreatic) collection and reduction of (peri)pancreatic fluid collections by <50% after APD—have not been described in any other reports for guiding PCD intervention.

## Limitations and Perspectives

In both our previous and present studies, we demonstrated that the novel step-up approach in addition to APD offers an interesting alternative to the present classical step-up strategy in selected cases of AP with lower morbidity and mortality. Nevertheless, this study has some limitations. First of all, this is a retrospective study, and some important data are lacking for a substantial number of patients. Secondly, the study represents the evolution of a step-up strategy for treating AP in our institution, especially because it is the first time that APD has been introduced as the second step in a novel step-up approach. Due to the effects of a learning curve, a lower rate of procedure-related complications may be expected with adequate experience and appropriate technique. Third, we only selected partial parameters for predicting the proper use of PCD. There is a wealth of other information regarding the validity and security of PCD, such as imaging parameters. Lastly, there is little experience reported for the details of PCD in the present study. To address the above questions, further prospective multicentre studies are necessary to confirm the validity of this new step-up approach and to define more effective and applicable factors that influence the efficacy of PCD.

Similar to other diseases, it is optimal to provide individualised guidance for PCD application in treating AP with fluid collections. Thus, more complicated information should be introduced and considered when setting predictors of PCD. For example, patients with a relatively high body mass index (BMI, ≥ 23) had more highly infected necrosis, infectious complications, more persistent organ failure, and higher mortality [38]. Thus, to effectively predict PCD intervention, it is advisable to exercise extra caution for patients with AP with a high BMI. Additionally, it is known that male and female patients differ in the development of AP. It has been shown that admission serum estradiol level (102 pg/ml) is a good marker of disease severity and is a predictor of mortality in patients with AP [39]. Therefore, we may use different indicators for PCD intervention when treating AP patients with high serum estradiol levels.

In addition to the timing of PCD, detailed information regarding PCD intervention is also very important. For example, the indication for the use of the correct percutaneous catheter for (peri)pancreatic fluid collections should be carefully considered. Some catheters are obviously not suitable and may lead to fatal outcomes. In our future studies, we will concentrate on specific details of PCD related to the viscosity of the fluid, location of fluid collections and number of fluid collections to optimise the results. In other words, we will sequentially target the optimal access, size and number of catheters, and timing for extracting the catheters. There is a trend toward computationally estimating the appropriate parameters of PCD and controlling each subtle performance parameter of PCD. The achievement of the above goal depends on the concerted efforts of a multidisciplinary team of interventional endoscopists, radiologists, intensivists and surgeons dedicated to the management of AP and its complications.
